# NSF DARE—transforming modeling in neurorehabilitation: a patient-in-the-loop framework

**DOI:** 10.1186/s12984-024-01318-9

**Published:** 2024-02-13

**Authors:** Joshua G. A. Cashaback, Jessica L. Allen, Amber Hsiao-Yang Chou, David J. Lin, Mark A. Price, Natalija K. Secerovic, Seungmoon Song, Haohan Zhang, Haylie L. Miller

**Affiliations:** 1 Biomedical Engineering, Mechanical Engineering, Kinesiology and Applied Physiology, Biome chanics and Movement Science Program, Interdisciplinary Neuroscience Graduate Program, University of Delaware, 540 S College Ave, Newark, DE 19711 USA; 2 Department of Mechanical Engineering, University of Florida, Gainesville, USA; 3 Electrical and Computer Engineering, University of Washington, Seattle, USA; 4 Division of Neurocritical Care and Stroke Service, Department of Neurology, Center for Neurotechnology and Neurorecovery, Massachusetts General Hospital, Harvard Medical School, Boston, USA; 5 Department of Veterans Affairs, Center for Neurorestoration and Neurotechnology, Rehabilitation Research and Development Service, Providence, USA; 6 Department of Mechanical and Industrial Engineering, Department of Kinesiology, University of Massachusetts Amherst, Amherst, USA; 7 School of Electrical Engineering, The Mihajlo Pupin Institute, University of Belgrade, Belgrade, Serbia; 8 Laboratory for Neuroengineering, Institute for Robotics and Intelligent Systems ETH Zürich, Zurich, Switzerland; 9 Mechanical and Industrial Engineering, Northeastern University, Boston, USA; 10 Department of Mechanical Engineering, University of Utah, Salt Lake City, USA; 11 School of Kinesiology, University of Michigan, 830 N University Ave, Ann Arbor, MI 48109 USA

**Keywords:** Computational modeling, Patient-in-the-loop, Sensorimotor adaptation, Digital Twin, Neuroplasticity, Musculoskeletal, Sensory, Pain, Neurological condition, Neurodevelopment

## Abstract

In 2023, the National Science Foundation (NSF) and the National Institute of Health (NIH) brought together engineers, scientists, and clinicians by sponsoring a conference on computational modelling in neurorehabiilitation. To facilitate multidisciplinary collaborations and improve patient care, in this perspective piece we identify *where* and *how* computational modelling can support neurorehabilitation. To address the where, we developed a patient-in-the-loop framework that uses multiple and/or continual measurements to update diagnostic and treatment model parameters, treatment type, and treatment prescription, with the goal of maximizing clinically-relevant functional outcomes. This patient-in-the-loop framework has several key features: (i) it includes diagnostic and treatment models, (ii) it is clinically-grounded with the International Classification of Functioning, Disability and Health (ICF) and patient involvement, (iii) it uses multiple or continual data measurements over time, and (iv) it is applicable to a range of neurological and neurodevelopmental conditions. To address the how, we identify state-of-the-art and highlight promising avenues of future research across the realms of sensorimotor adaptation, neuroplasticity, musculoskeletal, and sensory & pain computational modelling. We also discuss both the importance of and how to perform model validation, as well as challenges to overcome when implementing computational models within a clinical setting. The patient-in-the-loop approach offers a unifying framework to guide multidisciplinary collaboration between computational and clinical stakeholders in the field of neurorehabilitation.

## Introduction

Neurorehabilitation exemplifies how multiple disciplines—physical therapy, biomedical engineering, medicine, neuroscience, kinesiology, and others—come together with the common purpose of improving the lives of those with a neurological condition [1–5]. Yet major challenges for multidisciplinary research are differences in theory, approach, and terminology between disciplines [6, 7], all of which may hinder both collaboration and the development of improved neurorehabilitation. In this perspective piece, we propose a general, operational framework to promote synergistic collaboration among computational modellers, clinicians, and patients to improve functionality and quality of life.

Our proposed framework is built upon the International Classification of Functioning, Disability and Health (ICF) [8], a recent framework for precision rehabilitation [9], patient involvement in health care decision making [10], and works where computational models have been used to improve health care [11–14]. The ICF is the world health organization (WHO) framework to measure both disability and health of a patient, and is commonly utilized for rehabilitation management in clinical practice [8, 15–21]. Of particular interest here is the use of the ICF to identify a patient’s body structure and function, which can all be targeted during therapy to promote recovery or habilitation to a condition state [8]. Recently, a precision rehabilitation framework has been proposed that incorporates the IFC and high-fidelity data to, critically, continuously monitor a patient’s condition over time [9]. This precision rehabilitation framework also considers clinically important factors such as clinical phenotype, treatment type, and prescription. Further, patient involvement in clinical care has been shown to improve patient satisfaction and health, productivity of the service provider, and treatment outcome [22–28]. It is important to consider the aforementioned clinically grounded work when identifying where along a clinical pipeline that computational modelling can provide support to neurorehabilitation.

Computational modelling has proven particularly effective for drug delivery [11–14]. One well-known success story is the use of feedback control theory to deliver insulin in diabetics [11]. Continual monitoring of glucose levels and a computational model of both glucose and insulin dynamics are used to optimize insulin delivery through a pump. Critical to this approach is that the patient is directly within the control loop, where continual monitoring of glucose to determine optimal insulin delivery has been shown more effective than conventional treatment and leads to less diabetic complications [12]. This ‘patient-in-the-loop’ approach has also been proposed for anesthesia and HIV medicine [13, 14]. To improve mobility and functionality, there have been recent advances using a patient-in-the-loop approach in the areas of deep brain stimulation (DBS) for Parkinson’s disease [29, 30] and prosthetics for amputees [31, 32]. Our framework differs from these works and others [29–33], since our framework also jointly considers the ICF, diagnostic models, and patient involvement. Here we will consider how various sensorimotor adaptation, neuroplasticity, musculoskeletal, and sensory & pain models can be used within a patient-in-the-loop framework to facilitate neurorehabilitation.

We propose a comprehensive patient-in-the-loop framework that incorporates computational modelling, the ICF, continually monitoring a patient’s condition [9], and patient involvement to support neurorehabilitation. In particular, we will focus on where and how sensorimotor adaptation, neuroplasticity, musculoskeletal, and sensory computational models could be used to diagnose and optimize treatment for patients to improve their functionality and quality of life. Objectives of this perspective piece are (i) to create a patient-in-the-loop framework that identifies where along a clinical pipeline that computational modelling can support neurorehabilitation for a broad range of neurological and neurodevelopmental conditions, (ii) to identify current state-of-the-art and future directions in computational modelling that can support neurorehabilitation, and (iii) highlight the importance of model validation.

In the following sections, we will first address the patient-in-the-loop framework (ICF: patient body structure & function, data, diagnostic models & clinical decision-making by practitioner, intervention type, treatment models and prescription, recovery and habilitation, and patient involvement). We will then address types of models (data-driven and fundamental models), models for neurorehabilitation (sensorimotor adaptation & learning models, neuroplasticity models, musculoskeletal models, sensory and pain models), and model validation. Finally, we will discuss future challenges and provide a summary of this perspective piece.

## Patient-in-the-loop framework

**Fig. 1:**
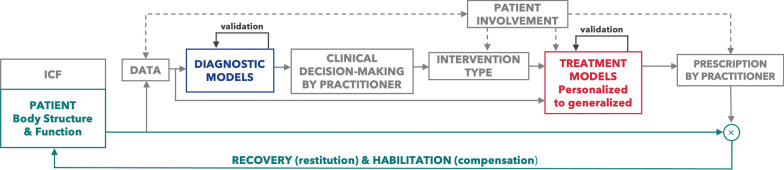
Patient-in-the-Loop Framework. This general framework promotes the use of the ICF, multiple or continuous data measurements over time, patient involvement, and computational models to aid clinicians in diagnosing and prescribing treatment. Specifically, within this ‘patient-in-the-loop’ framework, continual monitoring of a patient’s body structure and function allows for updates to diagnostic and treatment models that inform new and revised prescription by a practitioner to maximize recovery and habilitation. Both fundamental (e.g., motor adaptation, neuroplasticity, musculoskeletal, and sensory) and data-driven (e.g., machine learning) diagnostic and treatment models, which must be properly validated, can be utilized to better inform and facilitate more effective neurorehabilitation for a range of neurological and neurodevelopmental conditions

**Fig. 2:**
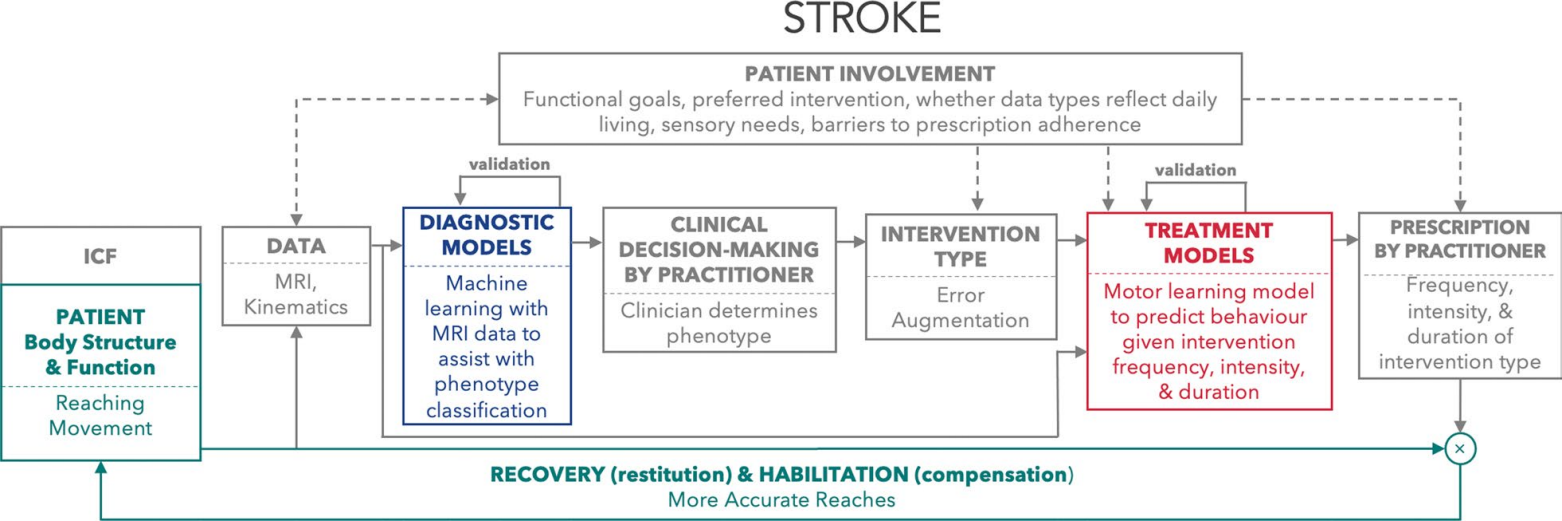
Computational models can be used within a patient-in-the-loop framework to improve post-stroke reaching. This example uses machine learning to assist with phenotype classification, error augmentation as the intervention type, and a sensorimotor learning model to better inform treatment prescription with the goal of improving reach accuracy during the recovery & habilitation process. Error augmentation magnifies visual and or haptic error signals, the difference between sensory feedback and an expected sensory target, which has been shown to improve both reaching behaviour and clinical measures of functionality for individuals post-stroke [114]. Patient involvement at several points within this loop is used to support model personalization, adherence, and tailoring of intervention plans to functional goals

**Fig. 3:**
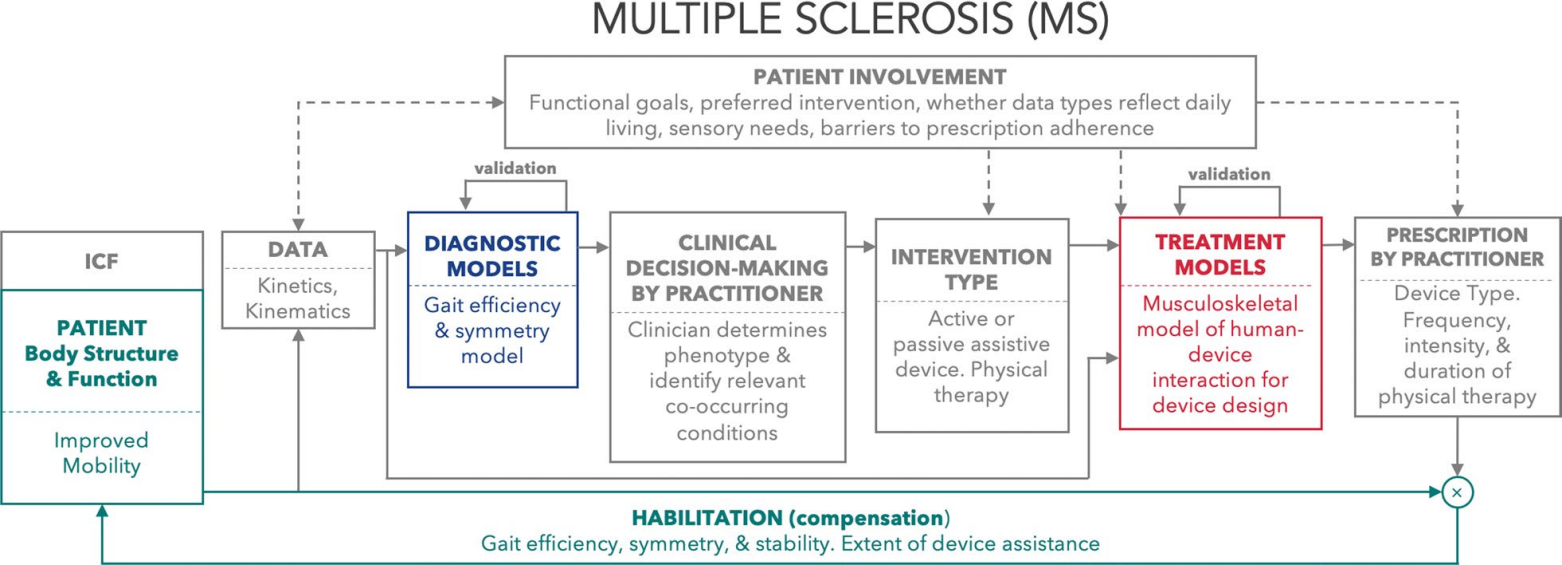
Here we use a worked example of multiple sclerosis within the patient-in-the-loop framework to improve mobility. Here musculoskeletal models of gait efficiency and symmetry are used to aid diagnosis, a device is used for intervention, and a musculoskeletal model of human-device interaction is used to predict several gait metrics when using the device. Patient involvement occurs at several points within the loop

**Fig. 4:**
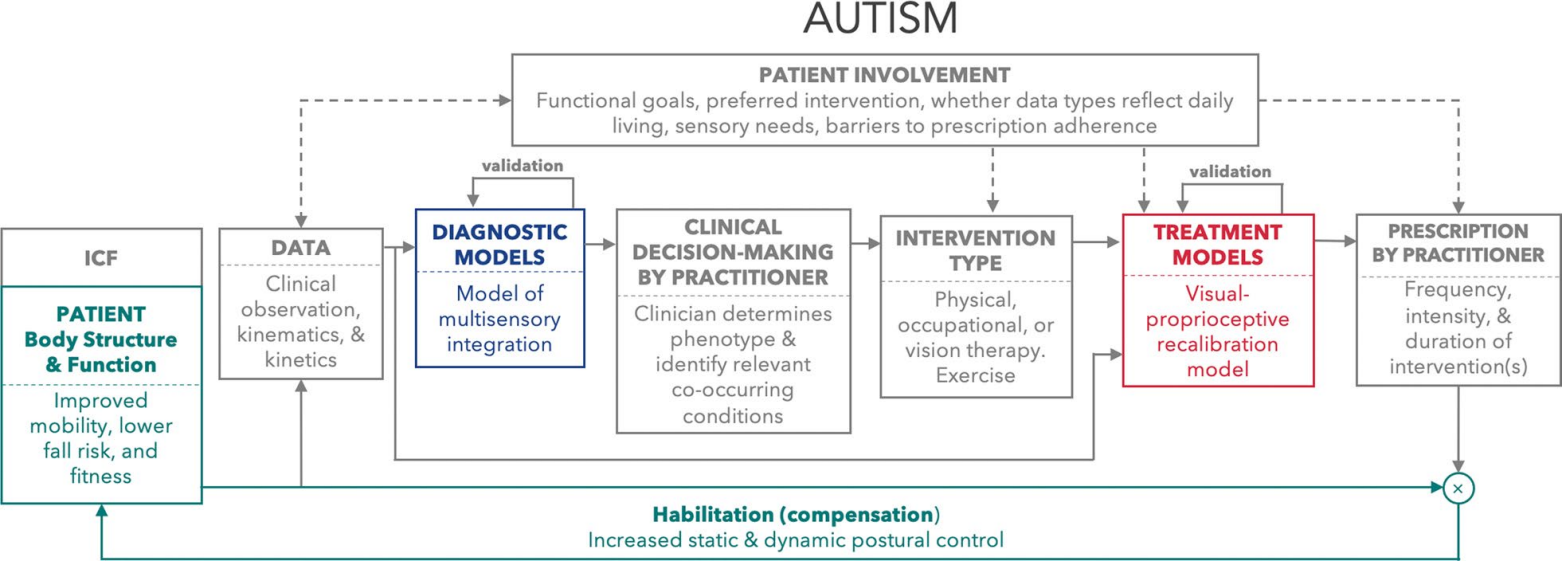
Computational models of multisensory integration and the oculomotor system can be applied to habilitation in autism. Patient involvement at each point in the loop can inform selection of data inputs, diagnostic and treatment model development and implementation, and selection of intervention type based on potential barriers to adherence, sensory needs, and personal functional goals. Treatment prescription can be informed via model predictions of visual-proprioceptive recalibration that account for developmental changes across the lifespan, with the goal of improving static and postural control to aid mobility, lower fall risk, and improve fitness

Our goal was to develop a patient-in-the-loop framework to identify where and how computational modelling can be implemented within clinical care to further support neurorehabilitation (Fig. 1). This framework considers the ICF, the use of multiple or continuous data measurements over time as in a recent precision rehabilitation framework [9], patient involvement, and computational models to support diagnostics and treatment prescription while continually assessing and optimizing the recovery and habilitation of a patient’s body structure and function. Our framework is intentionally general, in a similar vein to the ICF, so that it can be applied to a range of neurological and neurodevelopmental conditions (Figs. 2, 3, 4). Within this paper, we refer to computational models very generally as equation(s) that can be used to simulate some system or describe some phenomena, such as a dynamical system, control policy, adaptive process, a mapping between inputs and outputs, etc. We define diagnostic models and treatment models as any computational model that can be used to aid in the diagnosis or treatment prescription of a patient, respectively. Note that in some cases that the same computational model, such as one that describes the musculoskeletal system, may be used either as a diagnostic model or treatment model depending on the particular context that it is used. Directly below we expand on each component of this patient-in-the-loop framework.

### ICF: patient body structure and function

We begin the patient-in-the-loop framework with the ICF [8, 34]. The ICF is a well-known framework to guide therapy across a variety of domains [8, 15–21, 34], including neurorehabilitation for neural injury (e.g., Stroke [35]), movement conditions (e.g., Parkinson’s disease [36]), or neurodivergence (e.g., autism [37]). Here we focus on a patient’s *body structure and function* within the ICF, which are important to consider for computational modelling. While we apply the ICF in our patient-in-the-loop framework, we do not provide an exhaustive description of the ICF and would refer the reader to [34].

### Data

Our patient-in-the-loop framework promotes the use of monitoring patient data that relates to their body structure or function. These data can be collected continuously or at multiple time-points to support neurorehabilitation [9]. Such data can include kinematic and kinetic measurements of eye and body movement, electromyography (EMG) and electroencephalography (EEG) to respectively measure muscle and brain activity, medical imaging with ultrasound, X-ray, and magnetic resonance imaging (MRI), and clinical measures of functionality (e.g., Fugl-Meyer [38] and MDS-UPDRS [39]). Moreover, it is becoming increasingly possible to collect much of this data not only in laboratory settings, but also in the clinic and at home due to exponential improvements in wearable and portable sensors [40–44] and markerless video-based technologies [45, 46] in the past decade. Further, the low-cost and widespread use of wearable technologies may also enable collecting baseline data of an individual’s health and behaviour prior to the onset of a neurological condition. We envision that these advancements will enable researchers and clinicians to monitor patient condition in both controlled and naturalistic environments, as well as comparing data following the onset of a neurological condition to both normative measures across the lifespan and an individual’s own baseline.

### Diagnostic models and clinical decision-making by practitioner

Once patient data has been collected, computational models can assist a clinical practitioner with an initial or revised diagnosis of the patient condition. For example, a computational model may indicate the likelihood that a patient has a particular neurological condition based on kinematics [47] or neuroimaging [48]. It has been suggested that early disease onset detection can improve patient outcomes, such as in Parkinson’s disease [48]. Further, such models may also provide information as to whether it might be beneficial to try a different neurorehabilitation strategy. Below in "Models for neurorehabilitation", we discuss the use and potential of several computational models (sensorimotor adaptation, neuroplasticity, musculoskeletal, and sensory & pain) to aid in diagnosing a condition. An important consideration is ensuring that these models are properly validated, which we address in more detail below (see "Model validation"). Importantly, within the framework a trained medical practitioner (e.g., physical therapist, medical doctor, etc.) makes a diagnosis that is informed by a computational model.

### Intervention type

Once a medical practitioner has made a diagnosis informed by a diagnostic model, they are responsible for selecting the type of intervention used to treat the patient. Traditional therapy guided by a physical therapist or occupational therapist, often with drug management (e.g., L-Dopa for Parkinson’s disease [49]), remain the standard of care for neurological conditions. In addition to traditional approaches, technological advancements have and will continue to offer new alternatives that may further improve patient outcomes. Some common examples of different intervention types include robot-guided therapy [50–53], treadmill training [54–56], exoskeleton [57–59], orthotics [60–64], and brain-machine interfaces [65–68]. Whether receiving traditional therapy or interacting with a device, it is important to consider how a patient will adapt and respond to their particular prescribed treatment.

### Treatment models and prescription

Computational models can play an important role in the process of prescribing and monitoring outcomes of treatment for neurological conditions. For the purposes of this perspective piece, we will focus on sensorimotor adaptation, neuroplasticity, musculoskeletal, and sensory computational models. As an example, sensorimotor adaptation models describe how human motor behaviour changes over time in response to an environmental stimuli (e.g., external loads), and can be used to specify treatment duration that maximizes the retention of relearned, functional motor skills [69]. Further below, we expand on several different model types (i.e., machine learning, sensorimotor adaptation, neuroplasticity, musculoskeletal, and sensory & pain) and how they can be used to aid in treatment prescription. This manuscript primarily focuses on fundamental models that, in contrast to data-driven machine learning models, often generalize better since they attempt to understand and capture underlying mechanisms (see "Fundamental models"). It might be possible to use model simulations to generate synthetic data to make comparisons between the patient and outcomes at multiple point across the framework (e.g., diagnostic model outputs). Further, another potential approach would be comparing different treatment models at the stage of selecting the type of intervention [9]. That is, using models to simulate different intervention types may help guide the selection of intervention type.

A critical component of the proposed patient-in-the-loop framework, is the use of computational modelling for helping the medical practitioner in prescribing appropriate treatment. The FITT (frequency, intensity, time, and type) principle can be applied in this context [70]. Frequency is how often the treatment is performed, such as once per day. Intensity is the difficulty of a particular activity, such as maintaining some target level of heart-rate, walking speed, or load. Time is how long the treatment is performed, such as 30 min. Treatment type we have previously described above, which can include traditional therapy to interacting with some passive or powered device. Moreover, behavioural support for neurodivergent individuals can also fall under the FITT principle [71]. Computational modelling has the potential to provide insights into which type of treatment should be utilized, while optimizing the optimal frequency, intensity, and time to to maximize the recovery and habilitation of the patient’s body structure and function.

### Recovery (restitution) and habilitation (compensation)

Our patient-in-the-loop framework aims to prescribe treatment that promotes recovery or habilitation to patient body structure or body function (represented by the multiplication symbol in Fig. 1). As in Levin and colleagues, here we use the term recovery as improvements that result from restitution of biological structure and function [72]. The word habilitation represents a compensation to neurodivergence (e.g., autism) or a neurological condition (e.g., stroke). Habilitation is the use of different biological structures or function one would typically use, or the use of a passive or powered device, to carry out some desired activity. Habilitation as a term also encapsulates neurodivergent individuals, such as those with autism, where the goal is to improve outcomes since it is not possible to recover from a condition that someone is born with [73, 74]. However, it is important to consider that individuals will likely respond differently to the same treatment or may appear to have a different capacity to improve [75]. Moreover, computational models that prescribe treatment are unlikely to yield a perfect prediction of how a patient will respond to treatment prescription.

To address individual differences and imperfect model predictions, the proposed framework promotes the use of continual or multiple data measurements of body structure and function. *Crucially, continual or multiple data measurements over time allows for updates to treatment models parameters—thereby placing the ‘patient-in-the-loop’—so that treatment models can better predict and inform prescription that aims to maximize the recovery and habilitation of the patient’s body structure and function.*

### Patient involvement

The primary goal of the patient-in-the-loop framework is to utilize computational models to maximize recovery and habilitation of a patient’s body structure and function. This should ideally include continuous patient involvement throughout the processes of providing data, selecting intervention type, goals and constraints of models, and prescription. Patient involvement has been shown to improve treatment outcome, patient satisfaction, and health [22–28]. In line with the National Institutes of Health (NIH) recommendations [10], we encourage patient involvement with their care in decision-making at each step in their care trajectory, from first concern to intervention. As one would expect, the patient or their caregiver is responsible for consenting to and providing data to help make a diagnosis (dashed arrow from patient involvement to data; Fig. 1). The collected data and the interpretation of those data should also be shared with the patient (dashed arrow from data to patient involvement), so that they can be involved with subsequent clinical care decision-making. Once a patient is well-informed, they should be involved with both selecting the intervention type and the prescription. For example, the patient may have a preference for a particular treatment based on comfort levels, cost or time constraints, or several other personal factors. Further, it is possible to incorporate some of these personal constraints into computational modelling. For example, a patient’s constraints on the amount of time and frequency with which they can engage in physical therapy can be factored into sensorimotor adaptation models. Likewise, the patient may have their own personal goals on their desired outcomes and functionality, which may influence the intensity or type of the prescribed treatment. Patients can also inform the process of model development and feature selection, for example by reporting on their own experiences and internal states. This information adds value to development of diagnostic models by grounding them in patients’ lived experiences. Finally, the patient can continue to provide feedback on the treatment type and prescription in followup appointments, and be involved in decisions as to whether the care should continue as planned or if there should be changes to the treatment.

## Types of models

### Data-driven models

Data-driven computational modelling approaches are often used to identify patterns or relationships from data in an effort to drive neurorehabilitation efforts. In this paper and the sections below, we focus primarily on mechanistic models that aim to understand or capture the physiological processes underlying these relationships (see "Fundamental models"). However, purely data-driven models also hold significant potential to aid treatment prescription to estimate recovery and habilitation [76]. In contrast to mechanistic models, data-driven modelling approaches look for features in the recorded data (clinical, biomechanical, physiological, etc.) that are associated with and/or predictive of some meaningful outcome measure (e.g., functional impairment level, community participation, etc.) *without* modelling the underlying processes that produce those relationships. While there are some additional examples in the "Models for neurorehabilitation", here we provide a very brief overview of data-driven modelling efforts for neurorehabilitation.

Data-driven models for neurorehabilitation can generally be classified in two categories: (1) regression and classification approaches, and (2) deep learning approaches. The first step to regression and classification approaches is typically a data reduction process, in which high-dimensional data is transformed into a lower-dimensional representation via manual feature selection or algorithms such as principal component analysis [77–83]. This step helps to make the variability in complex continuous and/or multi-modal data amenable to interpretation and further analysis. The identified variables can then be used as input to regression, Bayesian, and classification models. For example, a recent study using regression approaches identified that younger ”brain age”, as measured from whole-brain structural neural imaging, was associated with better functional outcomes after a stroke [84]. Such a result points towards the importance of including brain age as a covariate in models predicting the response to neurorehabilitation. Classification models (e.g., support-vector machines, cluster analysis, etc.) that group data points based on their similarity have enabled the subdivision of stroke patients based on their underlying impairments [85–89], identifying potential targets for neurorehabilitation. However, relying on a lower-dimensional representation may limit the ability to capture subtle but important individual-specific patterns within the data underlying impaired function and/or improvements with rehabilitation. Deep learning approaches, on the other hand, that use the entire dataset instead of relying on a reduced representation, may better capture the diverse array of variables associated with biological processes that vary individually and change over time [90]. Such approaches have shown promise in making diagnoses, predicting treatment outcomes, providing real-time feedback, and guiding clinical decision-making [90–95]. Moreover, causal deep reinforcement learning to find an optimal dynamic treatment regime [9, 96] could be used to optimally select the best treatment type and prescription according to an optimal reinforcement learning control policy, which can be refined over time using continual data measurements.

Not only may data-driven models be useful for predicting individual-level treatment responses and provide insights intervention targets, they may also influence mechanistic, fundamental modelling efforts by serving as input to mechanistic, fundamental models. Moreover, relationships between data and outcome measures revealed via data-driven models may serve as motivation for developing novel mechanistic models to elucidate the physiological mechanisms underlying these relationships.

### Fundamental models

#### The role of fundamental modelling research in a patient-centered treatment paradigm

models are being developed not just as tools for clinicians, but as tools for building fundamental understanding of human motor control, adaptation, development, and impairment. While not always directly translational to a model-based treatment pipeline, this work is nonetheless essential for improving clinical outcomes. By improving our understanding of underlying mechanisms that cause neurological or behavioural changes, we improve the foundation for new treatments to be developed, whether or not the treatment directly involves a computational model. Conversely, without rigorous testing of our understanding of mechanisms that treatments exploit, we risk developing inaccurate models and ineffective, or worse, harmful treatment plans.

#### Mechanistic and phenomenological models

Mechanistic models explicitly describe the fundamental mechanisms that drive the relationship between observable inputs and outputs from first principles. Conversely, phenomenological models attempt to describe this relationship with a direct mapping from input to output (i.e., describing the “behaviour” of a system rather than lower-level contributions of its component parts).

Mechanistic models are capable of representing specific theoretical frameworks for a given mechanism. If a mechanism is theorized from first principles and is able to accurately and robustly describe the empirically observed relationship between input and output, it can be used to predict the response of the mechanism to novel interventions and new inputs. However, it is not practical or currently possible to derive every relationship between two phenomena down to the interaction between individual atoms, therefore mechanistic models are built with phenomenological components. Additionally, for describing processes within humans, while mechanistic models offer concrete theories that can be tested, it is often not possible to design experiments that can isolate the effect of a particular mechanism in humans. Therefore there is no guarantee that a mechanistic model can be confidently falsified even if it is incorrect. Despite these limitations, mechanistic models are important for neurorehabilitation because our understanding remains limited in many critical areas.

Phenomenological models are in many ways more practical when the question is not, “how does this system work?” but instead “what will the outcome be?”. Yet phenomenological models may not be as robust to novel circumstances as mechanistic models. That is, novel inputs may interact with internal mechanisms that did not significantly contribute to previously observed outputs. Nevertheless, phenomenological models are essential for model-based research with applications in neurorehabilitation. A well-characterized phenomenological model for a specific mechanism is often more useful in a practical sense than a mechanistic model of the same mechanism, because it allows for direct computation of the output in question. Rigorously validated phenomenological models are more likely to be directly translatable as practical tools for clinicians.

#### Using models to create new knowledge

In many situations, a model can be thought of as a formal representation of theory [97]. Such a model can be used to help design an experiment and test whether *a priori* model predictions are observed in the data [98, 99], or to investigate novel circumstances that have not been or cannot be experimentally tested [100]. Models can also be used to challenge prevailing interpretations of experimental work by demonstrating mechanistic validity of alternative interpretations [101]. If a model captures the mechanism of a particular neurological condition then it should be able to predict how an individual responds to treatment. If a model can reliably predict behaviour then it can also be used to optimize a treatment plan to promote recovery and habilitation. Next, we highlight how models of sensorimotor adaptation & learning, neuroplasticity, musculoskeletal, and sensory & pain can be used to support diagnostics and treatment.

## Models for neurorehabilitation

### Sensorimotor adaptation and learning models

In the interest of identifying unifying principles across the field of neurorehabilitation, we propose that adaptation is a universally-relevant treatment goal across a wide range of post-injury, neurodegenerative, and neurodevelopmental conditions. The ability of the sensorimotor system to adapt depends on the involved neural circuits and is critical to neurorehabilitation, where one must (re)learn functional motor skills to access a desired level of mobility and quality of life [2, 102]. In this section, we primarily focus on models of sensorimotor adaptation that characterize the biological process responsible for changes in motor behaviour over time. These models have been successfully applied to study common and functional motor skills, including reaching and gait. Adaptation models have been critical to understanding the learning principles of the sensorimotor system [103–106]. Understanding learning principles can be leveraged to determine the expected behavioural outcomes due to a neurological condition and aid in diagnosis, as well as predicting the behavioural response to treatment.

#### Sensorimotor adaptation and learning models to improve diagnosis

Studies that examine sensorimotor adaptation in individuals with a neurological condition may be useful for identifying disease biomarkers (e.g., kinematics, neural signals) and developing models that aid a medical practitioner to make a diagnosis. Reaching and gait paradigms have been used to study neurological conditions, including cerebellar ataxia, Parkinson’s disease, and Stroke. For example, during force-field reaching tasks [107], visuomotor rotation reaching tasks [108], and split-belt gait tasks [109] it has been shown that individuals with cerebellar ataxia have less ability to adapt their movement behaviour. Using the kinematic or kinetic data from similar adaptation paradigms could be used by a statistical or machine learning model, potentially in combination with other data (e.g., MRI, eye-tracking), to further inform a diagnosis. Some studies have fit a model separately to neurologically-atypical and neurologically-typical groups [104, 110], and in some instances accounting for medication status [111]. There are limited studies that have used adaptation models to explain a neurological condition [104], which is a very promising direction for future research.

#### Sensorimotor adaptation and learning models to improve treatment

An attractive feature of adaptation models is that they are dynamic and capture changes in sensorimotor behaviour over time [103–106], which could be leveraged by a medical practitioner to prescribe treatment. Predicting treatment outcomes represents an exciting opportunity for research. An important aspect of the patient-in-the-loop framework is the use of the FITT (frequency, intensity, time, and type) principle to prescribe treatment. Given some type of treatment, adaptation models could theoretically be used to find the optimal frequency, intensity, and time of some therapy to maximize patient outcomes. In terms of movement, these patient outcomes could range from generating straighter reaches [112–114] to producing more symmetric and energetically efficient gait [115–117]. Constraints, such as available time or physical effort, could also be factored into these optimization. Iterative collection of data over time in a patient-in-the-loop framework is necessary to continuously update and personalize model parameters to improve treatment predictions. Better treatment predictions may lead to more informed and effective treatment prescriptions that bolster recovery and habilitation.

#### State-of-the-art in sensorimotor adaptation and learning modelling

Sensorimotor adaptation can occur through several different mechanisms and their associated neural circuitry, including error-based, reinforcement, and use-dependent learning. Error-based adaptation has implicit and explicit components that reflect cerebellar processes [109, 118–122] and cognitive strategy [123–127], respectively. Error augmentation applies principles of error-based adaptation and has been shown as an effective approach to promote neurorehabilitation for those with stroke [112–114, 128, 129]. Error augmentation magnifies visual and or haptic error signals, the difference between sensory feedback and an expected sensory target, which has been shown to improve both reaching behaviour and clinical measures of functionality for individuals post-stroke [114]. In Fig. 2, we provide a worked example of the patient-in-the-loop framework that uses error augmentation as an intervention type and a model of sensorimotor adaptation to aid in prescribing treatment, with the goal of improving reaching accuracy for an individual post-stroke. Reinforcement-based processes promotes adaptation and neuroplasticity [98, 99, 106, 130–134], Reinforcement processes are linked to the dopaminergic system and basal ganglia, which become impaired with Parkinson’s disease [110, 135, 136]. Interestingly, reinforcement feedback can be used to promote sensorimotor adaptation for individuals with cerebellar ataxia, suggesting that intact reinforcement-based neural circuits can be exploited when there is damage to error-based neural circuits [104]. Use-dependent adaptation refers to the idea that the repetition of movements alone causes a change to the motor system [137, 138], perhaps through Hebbian-like processes (see "Neuroplasticity models" section below). Past research has focussed on isolating these different forms of adaptation [98, 139]. Understanding how these different adaptation processes interact [140], including when specific neural circuits are impacted by a neurological condition, will likely be a fruitful direction moving forward.

#### Future directions in sensorimotor adaptation and learning modelling to support neurorehabilitation

There are several promising future avenues where computational approaches may facilitate neurorehabilitation. Many of the aforementioned adaptation models have provided strong insights into learning principles and their respective neural circuitry [103, 104, 106, 133]. The majority of these models focus on short-term adaptation and retention, which is thought to reflect the refinement of an already existing motor controller [141]. However, neurorehabilitation occurs over long timescales where it is important to retain (re)learned behaviour over days, months, and even years. Motor learning and retention over these long timescales may align more with *de novo learning*, where there is thought to be a formation of a new motor controller [141–144]. An example of de novo learning is a child learning (and never forgetting) how to ride a bike (see [145]). More experiments and models are needed to better understand the principles that underlie de novo learning and its potential role in neurorehabilitation [145].

Another exciting opportunity for future research is to better understand how humans co-adapt with a device that aids mobility [146–149]. Wearable robotics that adapt to the human can lead to more energetically efficient gait [150, 151]. Yet further improvements to human-device interaction may be achievable by modelling the co-adaptation between a human and a device [152–154]. An important consideration for devices that (co)adapt is not to provide too much compensation and consequently limit the potential of true recovery by the patient. Rather in some instances it may be better for devices to utilize error augmentation techniques that amplify visual errors or haptic resistance, which has improved recovery for individuals post-stroke [114]. Several open questions remain on how two co-adapting agents interact, whether it be a human-device or therapist-patient, and how this knowledge can be used to support neurorehabilitation (Table 1).

**Table 1:** Sensorimotor adaptation and learning: state-of-the-art and future directions

State-of-the-art	a. Separate models for different adaptation processes: error, reinforcement,
	and use-dependency.
	b. Neural conditions and neural circuits associated with separate learning processes.
	c. Experiments and models that focus on short-term (i.e., single day) adaptation
	and retention.
Future directions	a. Studies and models that focus on the interaction of multiple learning processes,
	and exploiting these interacting and or redundant processes for neurorehabilitation.
	b. Research paradigms that focus on how motor controllers are constructed from
	scratch (de novo) to better understand long-term learning and retention to
	provide more effective and long-lasting patient outcomes.
	c. Develop models / devices that co-adapt and incorporate how humans learn,
	while optimizing assistance and resistance to maximize patient recovery.

### Neuroplasticity models

One of the underlying mechanisms of sensorimotor adaptation and learning is neuroplasticity. Broadly, the term applies to processes by which the nervous system changes, especially in response to experience [155]. Neuroplasticity is related to but not synonymous with the term ‘critical period’. A critical period specifically refers to times of heightened neuroplasticity during development or shortly following a neural injury. An example of a critical period following a neural injury is evident following a stroke. Even within the first few days following stroke there is heightened neuroplasticity, highlighting the importance of early neurorehabilitation [156].

#### Neuroplasticity models to improve diagnosis

Observations of neuroplasticity at the microscale, mesoscale, or behavioural level could be used to track disease state and update diagnoses. In an animal model of stroke, squirrel monkeys were given subtotal infarcts in hand representation of primary motor cortex [157]. Following subtotal infarcts, there were cortical map changes that were quantified by applying a microelectrode at successive sites in motor cortical area and measuring evoked movements in the upper extremity. These cortical map changes included relative expansion of motor areas subserving proximal forelimb into areas subserving distal forelimb. Although studies of this nature are commonly accepted as proof of neuroplasticity, there are limitations. Rigorous experiments and models must account for pre- and post-operative training and accurately represent human factors, such as relative lesion size and mapping conditions (e.g., during anesthesia vs. during movement).

#### Neuroplasticity models to improve treatment

Understanding the neuroplastic mechanisms is particularly important to identify precise intervention targets and monitor treatment response [158]. Noninvasive brain stimulation and human-device interaction are promising areas of investigation that tap into neuroplasticity with the goal of improving function after stroke [159].

The literature on efficacy of transcranial magnetic stimulation (TMS) has historically been mixed [160]. Smaller trials have shown efficacy [161, 162], while some large clinical trials have not shown efficacy [163]. It is possible that stimulation models would benefit from parameter optimization [164] to achieve a balance between generalizability and personalization. Excitatory TMS most likely potentiates residual circuitry to make concomitant training more effective [165] and represents a worthwhile avenue of future investigation.

Brain-machine interfaces and brain-computer interfaces have offered a promising approach for studying brain-behaviour relationships across scales in the context of neuroplasticity [166, 167]. The use of brain-machine interfaces and their corresponding algorithms to restore sensorimotor function has gained popularity in recent years for therapeutic purposes. Many promising results have been reported in neurorehabilitation, such as decoding communication for paralyzed people with tetraplegia and anarthria [168] as well as spinal cord injury [169]. These experimental outcomes primarily focus on restoring behavioural and motor functions at a behavioural level. Brain-machine interfaces also offer new avenues to understand neuroplasticity mechanisms that change brain connections at the neuron levels, making them particularly promising for rehabilitative purposes since the neurons involved in these interfaces are causally linked to behaviour. Prior studies have shown positive results, indicating that long-term brain-computer interface training could lead to stable neurocortical mapping [170] and enhance long-term cortical network connectivity [171].

#### State-of-the-art in neuroplasticity modelling

Neuroplasticity can be observed at multiple spatial scales: synaptic (i.e., microscale), mesoscale, and behavioural [172–174]. Perhaps, the most well-known type of plasticity is Hebbian plasticity, referring to the synaptic principle that “neurons that fire together, wire together” [175]. Hebbian plasticity has been extensively modelled and shown to be regulated by coordinated firing of pre- and post- synaptic neurons at millisecond time intervals, a phenomenon known as spike-time dependence and long-term potentiation [176]. Related concepts are homeostatic plasticity and long-term depression [177]. At the mesoscale, intracortical mapping studies and functional magnetic resonance show reliable changes in cortical areas in response to experience [157, 178–180] as observed with expert musicians [181–185]. Behaviourally, changes in measured behavioural variables are taken to represent changes in nervous system structure and function at the micro- and meso-scale [186]. Additionally, it is important to consider principles of neuroplasticity when developing or utilizing brain-machine interface algorithms that link different spatial scales.

#### Future directions in neuroplasticity to support neurorehabilitation

Here we identify several potential fruitful areas where neuroplasticity can facilitate neurorehabilitation. First, an attractive idea is for neuroplasticity models to aid in treatment planning. Ideally, one would develop a computational model of neuroplasticity that takes as input the intervention type and frequency of a prescribed treatment to predict functional outcomes. Before this can happen, mathematical equations of neuroplasticity need to be further developed. It will be important to define the specific scales at which these equations are operating, including synaptic, mesoscale, or phenotypic. Synaptic models of plasticity, such as spike-time dependent plasticity, have not been shown to generalize across more than one synapse or to the mesoscale or phenotype/behavioural outcome scales. As such, there is a need for computational models that characterize neuroplastic mechanisms across multiple spatial scales. Second, in addition to spatial scales, new models of neuroplasticity should also account for time scales and how they vary phenotypically within and between populations. The interaction among motor processes also likely changes over time and varies by phenotype. In upper extremity hemiparesis and recovery after stroke, there are multiple motor processes engaged, including reinforcement-based or error-based learning mechanisms [187]. While these processes are dissociable [98], their interaction across the time-course of recovery and habilitation has yet to be characterized. Third, brain-computer interfaces could aid in chronic stroke recovery by inducing neuroplasticity that changes brain connectivity [188]. However, a comprehensive understanding of how to engineer neuroplasticity through the design of brain-machine interfaces is still lacking. Exploring the potential to shape neuroplasticity through the design or adaptation of brain-machine interfaces represents a promising direction for future research [189]. Fourth, few studies have sought to understand the effects of movement itself on structural and functional neuroplasticity. Given that movement is a mainstay of treatment in neurorehabilitation (e.g., in the context of physical or occupational therapy), this is a promising avenue for investigation with high translational potential. Moving forward it will be important to link spatial and temporal neuroplastic changes to behaviour, and then target these neuroplastic-behavioural relationships with treatment to improve functional outcomes for those with neurological conditions (Table 2).

**Table 2:** Neuroplasticity: state-of-the-art and future directions

State-of-the-art	a. Hebbian learning models: spike-time dependence, long-term potentiation,
	homeostatic plasticity, and long-term depression
	b. Models at distinct spatial scales: microscale, mesoscale, phenotypic / behaviour.
	c. Brain-machine interface algorithms that relate neuronal activity to behaviour.
Future directions	a. Experiments and models that link the multiple spatial and temporal scales of
	neuroplasticity that can be used to developed informed and optimized treatment.
	b. Improving brain machine interface (BMI) design and developing brain machine
	interface algorithms that enhance neuroplasticity.
	c. More fundamental studies that examine understand the influence of movement
	on structural and functional neuroplasticity.

### Musculoskeletal models

Musculoskeletal models are computational representations of the biological musculoskeletal system, designed to simulate and analyze biological movements. By leveraging patients’ physical data (e.g., height, weight, age, strength, MRI, etc.) and movement data (e.g., kinematics and force/moments), customized models can be built to analyze recorded movements and estimate muscle activations and states (”tracking approach”) [190]. Additionally, musculoskeletal models, or neuromechanical models that also encompass neural control models in addition to musculoskeletal components, can be used to produce new motions in response to changes in the musculoskeletal system and environment, known as the ”predictive approach” [191, 192]. By using musculoskeletal models to track and predict movements, critical insights can be gained, providing timely diagnosis, personalized treatment for movement condition, and advanced understanding of biomechanics.

#### Musculoskeletal models to improve diagnosis

Musculoskeletal models are crucial for dissecting the root causes of neuromusculoskeletal conditions, quantifying their features, and communicating the nuances of these conditions to clinicians and patients. One promising application is to help identify underlying causes of impairments [116, 193, 194]. Once a model incorporating all potential neuromechanical factors of a patient is developed (e.g., muscle atrophy and sensorimotor noise), an ablation study can be performed (e.g., simulate with the potential factors one-by-one) to identify the most critical factors that most likely contributed to the impairment. Treatment plans can then be tailored more precisely, potentially leading to more effective interventions. Another potential use of musculoskeletal and neuromechanical models is to provide clinicians with a comprehensive quantification and visualization of a patient’s neuromechanics of movement, offering a detailed depiction of aspects such as joint movement [195], muscle length and force [196], muscle activations [197] and synergies [198, 199], and energy consumption [200]. Using these enhanced analyses on a patient’s movement could improve both clinicians’ and patients’ understanding of the impairments, clarifying the rationale behind prescribed treatments. These models can also be used to quantify the severity of movement abnormalities, complementing clinical metrics that are often subjective (e.g., Fugl-Meyer score [38]).

#### Musculoskeletal models to improve treatment

Musculoskeletal and neuromechanical models can also be used in the design, outcome prediction, and monitoring of various treatment modalities for movement conditions. For example, potential effects of physical therapy and exercise interventions can be simulated with these models [201, 202], leading to more precise implementation of targeted interventions that promote neuroplasticity and adaptation. Musculoskeletal simulations can also help surgeons to predict the potential impact of different surgical strategies on musculoskeletal outcomes [203, 204]. Furthermore, assistive device design (e.g., exoskeletons, prostheses, braces) can benefit from musculoskeletal models by analyzing and predicting interactions between the devices and the human body [205–207]. Musculoskeletal models can estimate patient body states in real-time, providing a basis for the control of assistive devices [208–210]. Musculoskeletal models can also facilitate the monitoring of rehabilitation progress, enabling clinicians to objectively assess the effectiveness of interventions and track patient improvement over time.

#### State-of-the-art in musculoskeletal modelling

Over the last two decades, musculoskeletal and neuromechanical modelling has advanced significantly. Initially focused on tracking approaches to estimate joint dynamics and muscle activations [190, 211], the field is now embracing predictive methodologies to study motion variations under different musculoskeletal and environmental conditions [191, 192]. These models have enriched understanding of human movement and musculoskeletal conditions, shedding light on injury mechanisms like ACL ruptures [212] and ankle sprains [213], and aiding clinical decisions in osteoarthritis cases by assessing joint load [214, 215]. They have been used in evaluating surgical outcomes, such as hamstring lengthening surgery for children with crouch gait [216], and have helped characterize the impacts of musculoskeletal changes on movement patterns in aging [193], muscle atrophy [194, 217], and hemiparetic gait [116]. Moreover, these models are being applied in developing exoskeletons and prostheses [206, 218–220], and integrated into real-time controllers for assistive devices [209, 218, 221, 222].

#### Future directions in musculoskeletal modelling to support neurorehabilitation

While primarily used to study and evaluate musculoskeletal conditions today, musculoskeletal models are poised for substantial progress that could dramatically enhance their precision and clinical relevance in neurorehabilitation. As shown in the worked example of the patient-in-the-loop framework in **Figure 3**, musculoskeletal models could be employed to customize physical therapy programs and the design or prescription of assistive devices. This customization would be specifically tailored to an individual patient’s mobility level, clinical phenotype, and rehabilitation goals, thus underlining the pivotal role of these models in future neurorehabilitation strategies.

Model customization is necessary to capture patient-specific musculoskeletal features for diagnosis and treatment, possibly through the use of MRI, ultrasound, dynamometry, and motion data. Despite the importance of developing neuromechanical control and learning models that can predict human behaviour, it remains an immense challenge largely due to our limited understanding of the nervous system. Here we highlight potentially valuable directions. First, it will be important to refine objective functions to generate more reliable and accurate predictions. Current research predominantly utilizes optimality principles based on metabolic energy and muscle activation to produce predictions. However, it will be valuable to explore other physiologically-plausible objectives, such as stability [223, 224], fatigue [225], and comfort [226]. Second, more advanced physiologically-plausible control models [227–230] are necessary, extending beyond the current focus on locomotion to include a broader range of movements. The integration of advanced machine learning techniques, such as deep reinforcement learning, could be critical in training controllers to produce this more diverse range of movements [192]. Third, the inclusion of further neurological details, such as sensory dynamics, activation dynamics, and the properties of neural activation, could make our neuromechanical models more congruent with actual neural activity recordings [231], thereby enhancing their realism and applicability. Fourth, incorporating sensorimotor adaptation and learning models into neuromechanical simulations holds potential for advancing neurorehabilitation modelling. Such incorporations could show how musculoskeletal alterations, assistive devices, or interventions may induce movement adaptations. Fifth, adapting these control models to accommodate neural pathologies represents both a significant challenge and an indispensable step towards enhancing their relevance in neurorehabilitation (Table 3). Collectively, these advancements could be complemented by rigorous experimental validation involving human experiments [232]. Importantly, collaboration with clinicians is necessary to ensure that any advanced models tools are developed and utilized to meet the requirements of clinical practice and patient care.

**Table 3:** Musculoskeletal: state-of-the-art and future directions

State-of-the-art	a. Predictive models of motion under different musculoskeletal and environmental
	conditions, ranging from changes due to age and walking over uneven terrains.
	b. Modelling musculoskeletal conditions and injury, such as osteoarthritis.
	c. Evaluating surgical outcomes, such as hamstring lengthening for crouch gait.
	d. Modelling human-assistive device interactions with integrated real-time control.
Future directions	a. Consider plausible objectives in cost functions that relate to patient goals,
	including stability, fatigue, and comfort.
	b. Inclusion of neurological details that become impacted with neurological
	conditions, such as sensory feedback and other neural dynamics.
	c. Incorporating learning and neuroplasticity principles, which are important
	when recovering from a neural condition or interacting with an assistive device.
	d. Advancing models to accommodate various neural condition and injury.

### Sensory and pain models

Mathematical and computational techniques can be used to simulate processes underlying sensory perception and pain sensation. These models aim to capture the complex interactions between various physiological, neural, and cognitive factors across a wide range of conditions including neuropathic pain, migraine, autism, and Parkinsonism. Sensory models can inform the development of better diagnostic tools and treatment approaches, including advanced neuroprosthetic devices. Computational modelling offers a less-invasive means of generating new knowledge on human sensation and pain compared to in vivo experimental paradigms. Yet, such models have been based on neurotypical function and development, neglecting to characterize atypical acquisition, processing, integration, and use of sensory information. This has limited their application in clinical research and in diagnosis and management of conditions with known sensory processing differences.

#### Sensory models to improve diagnosis

Current diagnostic practice for atypical sensory function in neurodevelopmental and neurodegenerative conditions relies heavily on clinician observation (e.g., Romberg’s Test [233]), patient self-report (e.g., Adolescent/Adult Sensory Profile [234]) or standardized assessments with binary or ternary scoring systems (e.g., Mini-BEST [235]). We have yet to comprehensively model the complexities of atypical sensory processing and their manifestation within or between clinical populations, and across development. For example, a clinician may observe similar postural instability and sensitivity to auditory stimuli in a patient diagnosed with superior canal dehiscence syndrome and a patient diagnosed with autism, but the two have very different neurobiology and require different management of their sensory experiences.

Visuomotor integration is a promising target for computational modelling to characterize the link between behaviour and neural mechanisms. Sensory weighting for postural control and movement is heavily reliant on visual input in early and middle childhood, and more adaptable in adolescence [236–238]. [239]. Neurotypical adults maintain the ability to reweigh sensory information based on its integrity or availability but return to visual over-dependence with aging, which increases their risk of falls [240]. However, neurodevelopmental conditions like developmental coordination condition and autism may not exhibit typical sensory reweighing in some contexts [241–244]. Condition-specific models could offer precise diagnostic tools for distinguishing typical from atypical development and differentiating between neurodevelopmental conditions.

Recent advances in human-device interaction also support precise diagnosis of atypical sensory function. For example, to assess proprioceptive integrity following stroke or in response to perturbation [52, 245–248]. To optimize diagnostic and treatment applications of computational modelling, we must address questions of model sensitivity and specificity. Both condition-specific and general models must characterize the functional and temporal interplay between sensory systems [249], given the known importance of multisensory processing.

#### Sensory models to improve treatment

Visuo-proprioceptive recalibration represents a promising intervention type to improve sensory function, as evidenced in the reaching literature [250–253]. Computational modelling has been used recently to explain this sensorimotor adaptation process [254]. Visuo-proprioceptive feedback training shows promise for fall prevention in healthy aging [255] and improvement of bradykinesia in Parkinsonism [256]. Recent work suggests that proprioceptive, but perhaps not visual, recalibration is retained after remapping [257]. Computational modelling can be used to determine whether sensory recalibration potential differs between or within populations, enabling clinicians to predict who may benefit most from this approach.

Computational models personalized to patient-specific factors can predict the success of adaptation to neuroprosthetics, as seen in cochlear implant development [258]. As users adapt to implants, sensory remapping improves speech perception [5]. This process takes time and can lead to frustration or disuse. Acoustic models that drive cochlear implants are constrained by the number and tuning of channels, temporal resolution, and topographical discrepancy between the frequency information provided by the electrode and the natural tuning of the cochlea. Interactive genetic algorithms and evolutionary algorithms can support optimization of acoustic models at a more efficient rate of convergence than natural adaptation [259, 260]. Models simulating listener errors can also be used to inform development of such algorithms [258]. Demonstrating the utility of integrating models into a patient-in-the-loop framework, self-selected acoustic models for cochlear implants tuned to individual parameters via method-of-adjustment outperform high-dimensional models [261]. This approach highlights the need for a balance between efficient, generalizable models, and those personalized to account for individual features to optimize the efficiency and quality of rehabilitation.

#### State-of-the-art in sensory modelling

In the recent past, researchers focused on mimicking the natural coding of touch and integrating them into bionic devices in form of so-called biomimetic paradigms [262, 263]. To replicate the reality, receptor and afferent responses are simulated to estimate how mechanical input will be transformed into afferent spike trains and transmitted to higher-order somatosensory areas [264, 265]. These models offer a better understanding of touch afferent activation, as well as overcoming a major limitation of direct electrophysiological measurements which require stationary conditions, enabling analysis of activity in situations like walking or jogging. Similar models are also used to mimic the response of proprioceptors [266, 267], providing a novel guideline for controlling sensory restoration. Improvement of these models depends on the availability of large human and animal datasets containing electrophysiological recordings of afferent responses across a variety of different types of stimulation.

Mathematical representations of pain can guide development of hypotheses about underlying intervention targets. Most established computational models of pain focus primarily on binary prediction of pain perception using machine learning algorithms [268, 269]. These approaches typically fail to account for the specific features driving pain perception [270]. Computational models can also be used to understand and predict the pain perception-action cycle [271]. These models describe the dynamic interaction between the sensory experience of pain and the behavioural response [272, 273]. Most established models of pain are data-driven and based on neural networks [274–278]. By contrast, to our knowledge, there are relatively few hypothesis-driven, mechanistic models of pain.

#### Future directions in sensory modelling to support neurorehabilitation

The field of neurorehabilitation is faced with several remaining challenges in pursuit of robust, comprehensive sensory models. Models of motor control based on neurotypical development may not capture critical variability in sensory information acquisition. For example, atypical oculomotor control is a feature of many conditions (e.g., autism, schizophrenia, Parkinsonism) that poses challenges for the development of robust eye movement models. Atypical postural control is also a feature of many conditions that poses challenges for the development of generalizable models of body movement. Comprehensive multisensory models are essential for advancing technologies used in research and clinical contexts to quantify sensory processing in more naturalistic, less-invasive contexts.

Sensory experiences are highly subjective and influenced by psychological factors such as expectation [279], catastrophizing [280], optimization to the environment and task [281], and co-occurring conditions [282]. Pain experiences are also subject to the influence of contextual, social, psychological, and cultural factors [283]. These factors are often neglected or poorly operationalized in physiological studies. Mixed-methods approaches can be used to characterize the human sensory-perceptual experience, as qualitative responses can help to contextualize physiological and behavioural data [284]. Models that account for the full complexity of sensation and pain perception are needed to support development of comprehensive pain management strategies that address all relevant dimensions in concert (Table 4).

The proposed patient-in-the-loop framework is a means of ensuring that lived experiences and psychological/perceptual influences on sensory and pain processes are not lost in assessment and management. Fig. 4 offers a worked example of applying this framework to specific visuomotor neurohabilitation goals in autism, though certainly it has many other habilitation and rehabilitation applications. As in other domains of modelling, it is crucial to achieve a balance between data-driven and hypothesis-driven approaches to ensure that resulting knowledge is theoretically-productive and interpretable. Focus should be directed to development of models that offer new information about the relative importance of specific features used to predict or explain clinically-relevant outcomes [285], while factoring patient goals and constraints.

**Table 4:** Sensory and pain: state-of-the-art and future directions

State-of-the-art	a. Separate models for typical development and for specific neurodevelopmental
	and neurodegenerative conditions.
	b. Experimental paradigms and models that constrain dimensionality.
Future directions	a. Multisensory models that consider feedforward and feedback mechanisms.
	b. Sensitive and specific diagnostic models that account for general population
	variability and phenotypic heterogeneity within conditions.
	c. High-dimensional diagnostic and treatment models that account for interplay
	between physiological, cognitive, emotional, and behavioral factors to support
	optimization and personalization.

## Model validation

Modelling across all domains depends on rigorous validation to render models reliable as diagnostic, treatment, or research tools. Unification of validation processes across the field of neurorehabilitation is crucial to maximize the validity and translational potential of computational modelling. Best practice guidelines proposed for musculoskeletal model validation [286] could be adapted and extended to also accommodate models of adaptation, neuroplasticity, and sensation/perception.

### Validation targets

Determining the validity of a model entails evaluating: 1. Is the model feasible (e.g., does the fundamental model appropriately represent the hypothesis it aims to capture)?, and 2. Is the model accurate (i.e., does the model correctly reproduce the input–output relationship observed in the real world)? Feasibility must be satisfied before any meaningful interpretation of model behaviour can be made. Otherwise, model outputs do not reflect the hypothesized relationship, regardless of how well they may fit experimental data. Model accuracy must be evaluated against experimental observations. Models can be adjusted or reconceptualized to reduce model error (the difference between model-generated outputs and experimentally observed outputs), or discarded if experiments indicate no relationship between the hypothesis represented by the model and the observed behaviour.

The use of robust statistics and obtaining confidence intervals of parameter values are useful for model fitting. An absolute loss function, specifically the absolute difference between model-generated outputs and experimentally observed outputs, is known to be more robust than squared error since it is less sensitive to outliers [98, 287–289]. Once a loss function is selected, it is useful to find the confidence intervals of each model parameter. Bootstrapping is a resampling technique that can provide a nonparametric confidence interval for each model parameter [290]. Another benefit of bootstrapping is that statistical inference can be performed between different models since the confidence intervals are known [99, 106]. For example, one can calculate the probability that a neurotypical group will have significantly different model parameters than those with a neurological condition.

### Goodness of fit versus overfitting

It is also important to consider, however, that perfectly matching a set of observed behaviours is not the only purpose of a model. Instead, a useful model captures the critical dynamics between phenomena that drive most of the change in output given some change in input. Identifying the model parameters that dominate input–output dynamics is possible through sensitivity and uncertainty analyses [291], which generally involve perturbing model inputs in sequence and measuring the output response.

To find the simplest model that best explains the data and prevent overfitting an over-parameterized model to noise [292], tools such as AIC (Akaike Information Criterion) and BIC (Bayesian Information Criterion) consider the quality of the fit while penalizing the number of free parameters. Further, BIC and AIC can also provide insight into which of several theories, each represented by a model, is more likely to be true [99, 292, 293]. Cross validation is another useful approach to prevent overfitting and to test model accuracy. Cross validation uses multiple different subsets of the original data to fit the data, and then tests the resulting model accuracy on the remaining subsets of the data [294, 295]. This approach is particularly popular for machine learning and is useful for testing how accurate a model is when applied to new data sets. Utilizing models that are not overfit and generalize to new data sets will be particularly important when attempting to diagnose or prescribe treatment.

### Future factors to consider for model validation

Adherence to rigorous model validation is a critical step for ensuring that the development of a model-assisted neurorehabilitation paradigm will be appropriately robust. Ensuring that experiments are sufficiently powered is an important factor not only for reproducibility, but also for testing theories and model validation [296–300]. Model validation efforts should be supported by standardized criteria that are valid across the spectrum of proposed model scope, from population-level models to fully individualized “digital twins”. Clinical acceptance of computational models in a clinical rehabilitation framework will likely be greatly aided by such an effort to reliably demonstrate that these models are trustworthy. It is the responsibility of computational modellers to ensure that models do not impede the ability of clinicians or patients to make informed decisions, but rather strengthen it.

## Challenges

In addition to the model-specific challenges already discussed, there also exist challenges related to the data which serve as the input to all models described above. The access to high-dimensional, multi-modal data collected from ecologically-valid situations represents a potential paradigm shift in neurorehabilitation. However, there are several open challenges that must first be addressed to use this data effectively within computational modelling for neurorehabilitation. First, it is critical to identify what data is needed for each model. This involves identifying primary outcome measure(s) of interest (e.g., walking speed, community participation, etc.) and the physiological, biomechanical, and or clinical variables that are most relevant to that outcome measure. This step can require large samples of diverse patients to overcome the heterogeneity within a population (e.g., stroke, Parkinson’s disease, etc.), often necessitating multi-site collaboration. Thus, the second challenge is to overcome the potential inconsistency in data quality or availability across locations, people, days, etc. For example, MRI imaging quality can vary depending on scanner type, strength, pulse sequences, etc. A model that overcomes this variability will have the most potential for impact on neurorehabilitation [301]. In a similar vein, wearable sensor data to monitor movement can be sensitive to sensor placement and orientation [302–304]. Thus, models that rely on wearable sensor data must account for such variability to achieve optimal performance. Third, it is critical to carefully assess the performance of group-level statistics versus individualized data for model predictions. Or in other words, how much model personalization is necessary. In some cases, group-level statistics may be sufficient. For example, many studies have successfully grouped stroke patients based on their similarity in clinical, biomechanical, and/or physiological impairments [85, 86, 88, 89]. Do patients within each of these groups respond similarly to rehabilitation? If so, then models based on group-level statistics may be sufficient. In other cases, however, personalization may be critical. For example, personalized neural control models are able to better predict the immediate response to surgery in children with cerebral palsy [305] and stroke gait under novel conditions [306] than group-level and generic models. Determining the optimal level of personalization for any model will require careful assessment and evaluation of the extent to which additional and meaningful predictive capabilities are gained with increasing personalization. Lastly, developing models that rely on multiple time-point and/or continuous monitoring of multi-modal data in real-world settings require careful processing and advanced modelling techniques to analyze the large data sets that are generated. Deep learning methods that use the entire dataset instead of a reduced representation show promise in capturing subtle but important individual-specific patterns, which can guide clinical decision making via purely data-driven modelling approaches or serve as input into mechanistic, fundamental models. However, there is still substantial work to be done to guarantee the accuracy of these models in capturing the heterogeneity present across individuals and their utility in predicting response to neurorehabilitation.

We believe that implementing computational modelling within the clinical setting is a fruitful goal to strive towards. Yet there are several potential challenges, some of which we briefly mention here. There will likely have to be agreement on some of the tasks used to collect patient data [307], so that there is consistent and a sufficient amount of data to properly validate models. As mentioned above, it is imperative to ensure models are properly validated and viable before implementing to inform diagnostics and treatment. From a modelling perspective, many of the computation models have been designed and validated for very specific scenarios (e.g., walking in a straight line at a steady velocity, or reaching towards a target while moving the arm only in the horizontal plane). It will be important to develop models that generalize and capture more naturalistic movements that are relevant to the functionality of a patient. As mentioned in the introduction, a major challenge is getting researchers from multiple disciplines (engineerings, clinicians, etc.) to productively work together towards a common goal, despite often very different approaches and skillsets [6, 7]. To facilitate these collaborations, educational and technical workshops aimed at both clinicians and computational modellers would be beneficial to better understand one another’s theory, approach, and terminology. Increased federal funding towards (i) providing educational and technical workshops for modellers and clinicians, (ii) conferences that focus on computational modelling in neurorehabiliation that is attended by both clinicians and modellers, and (iii) grants that support collaboration between clinicians and computational modellers with the goal of improving neurorehabilitation would help to overcome many of these challenges.

## Summary and moving forward

We have proposed where and how computational modelling can be used to aid clinical diagnosis and treatment within our proposed patient-in-the-loop framework. In this perspective piece, we identified current state-of-the-art and highlighted potentially fruitful avenues of computational modelling across sensorimotor adaptation (e.g., co-adaptation with a device), neuroplasticity (e.g., linking spatial scales of neurons), musculoskeletal (e.g., stability), sensory (e.g., pain perception), and machine learning (e.g., optimal dynamic treatment regime [9, 96]) that can be used to support neurorehabilitation. It is imperative to validate these models before using them to help inform diagnosis and treatment. We believe that it will be equally important to take both mechanistic or patient-centered approaches that respectively consider underlying processes and functional outcomes, to eventually provide causal links along the continuum between neurons and behaviour.

Our patient-in-the-loop framework provides a guide to facilitate multidisciplinary research to improve patient recovery and habilitation. Our framework is intentionally general, in a similar vein to the ICF that it incorporates, so that it can be applied to a broad range of neurological diseases (e.g., Stroke, multiple sclerosis, etc.) and neurodevelopment conditions (e.g., autism). It incorporates the idea of multiple and or continuous data measurements of a patient’s condition over time, as described in a recent precision rehabilitation framework [9]. Further, this framework includes patient involvement throughout the process, from data collection to prescribed treatment, which has shown to improve patient satisfaction, treatment outcome, and health [22–28]. Importantly, this patient-in-the-loop framework identifies where and how computational modelling can support neurorehabilitation within a clinical pipeline. We encourage clinicians and modellers to utilize or adapt this patient-in-the-loop framework to develop informed and effective neurorehabilitation.

## Data Availability

Not applicable.
